# Characterizing Foveal Hypoplasia Using Optical Coherence Tomography Angiography: Evaluation of Microvascular Abnormalities and Clinical Significance

**DOI:** 10.3390/jcm12154992

**Published:** 2023-07-29

**Authors:** Jens Julian Storp, Julian Alexander Zimmermann, Moritz Fabian Danzer, Maged Alnawaiseh, Nicole Eter, Sami Al-Nawaiseh

**Affiliations:** 1 Department of Ophthalmology, University of Muenster Medical Center, 48149 Muenster, Germany; julian.zimmermann@ukmuenster.de (J.A.Z.); nicole.eter@ukmuenster.de (N.E.); s-alnawaiseh@outlook.de (S.A.-N.); 2 Institute of Biostatistics and Clinical Research, University of Muenster, 48149 Muenster, Germany; moritzfabian.danzer@ukmuenster.de; 3 Department of Ophthalmology, Klinikum Bielefeld gem. GmbH, 33604 Bielefeld, Germany; maged.alnawaiseh@klinikumbielefeld.de

**Keywords:** angiogram, FAZ, flow density, fovea plana, foveal avascular zone, foveal pit, thickness, vessel density, visual acuity, volume

## Abstract

This study aimed to evaluate foveal avascular zone (FAZ) features and macular flow density (FD) in various retinal layers in a cohort of patients with foveal hypoplasia (FH) using optical coherence tomography angiography (OCTA), in order to characterize microvascular abnormalities and explore their potential clinical significance. FAZ parameters and FD, as well as retinal thickness and volume values were analyzed and compared between patients with FH and an age- and gender-matched control cohort. Correlations between disease severity and visual acuity (VA), as well as between disease severity and FAZ features were evaluated. A total of 19 eyes with FH and 19 control eyes were included. The study group showed significantly higher FD values in the foveal sectors of the superficial and deep capillary plexus compared to controls. FAZ area, perimeter, and acircularity index (ACI) were noticeably altered in eyes with FH; however, they did not correlate with disease severity. Visual acuity was negatively correlated with disease severity. The results of this study provide evidence of altered microvasculature architecture specifically in the foveal sectors of patients with FH. The higher FD values in the foveal sectors of FH patients suggest a potential compensatory response of the retinal microvasculature. FAZ parameters and FD values of the foveal sectors could be used as part of an OCTA-based grading system in FH patients.

## 1. Introduction

Foveal hypoplasia (FH) is an uncommon congenital condition characterized by the underdevelopment or absence of the fovea. In humans, the fovea centralis constitutes the region of highest visual acuity. Within the fovea centralis lies the foveola, a depression in the macula’s surface, housing the highest concentration of cones in the photoreceptor layer. As this area lacks retinal vessels, it is referred to as the foveal avascular zone (FAZ). 

FH can manifest as an isolated condition or in conjunction with other clinical presentations, such as albinism, aniridia, retinopathy of prematurity, incontinentia pigmenti, achromatopsia, optic nerve hypoplasia, familial exudative vitreoretinopathy, congenital retinal macrovessels, and Stickler syndrome [1,2,3,4]. The clinical profile of FH often encompasses nystagmus and variable visual acuity.

Diagnosing FH involves assessing various factors including occasional pigmentary changes, absence of a funduscopic foveal light reflex, and evaluation through imaging modalities like fluorescein angiography, fundus autofluorescence, and optical coherence tomography (OCT). Particularly, OCT contributes to a more comprehensive understanding of the structural aspects and anatomical classifications of the foveal region.

Optical coherence tomography angiography (OCTA) has emerged as a non-invasive imaging modality, allowing for the quantification of retinal vasculature through the measurement of flow density (FD) and FAZ parameters while at the same time providing cross-sectional retinal images [5]. This technology has proven particularly valuable in characterizing diseases associated with altered retinal microvasculature [6,7,8]. Understanding the microvascular alterations in the retinas of individuals with FH is crucial for evaluating the underlying pathophysiological mechanisms of this condition. While previous studies have explored structural and functional abnormalities in FH, limited research has focused on specific changes in retinal FD and FAZ parameters in OCTA imaging. Due to the relative rarity of FH and the novelty of OCTA technology, most studies reporting on changes in microvascular parameters in FH patients have been limited to case series, depicting qualitative rather than quantitative data [9,10,11,12,13,14,15,16,17]. The studies which report quantitative data have demonstrated that while FH is associated with a reduction in FD in whole en face scans, it shows increased FD values in the foveal sector in the inner retinal plexus [18,19]. These reports, however, restrict themselves to the analysis of the entire macular scan and foveal sector. Further macular sublocations, deeper retinal layers and quantifiable morphological parameters of the FAZ have not been broadly covered in the literature to date. It is crucial to precisely understand the vascular changes associated with FH, as it has the potential to enhance our knowledge of disease pathogenesis and inform prognosis and future treatment strategies.

Thus, this study aims to evaluate macular FD and FAZ features in various retinal layers in FH using OCTA. The main aim of this study is to quantify vascular changes in different retinal layers in eyes with FH in order to characterize the microvascular abnormalities associated with this condition and explore their potential clinical significance. Additionally, we report thickness and volume values for individual retinal layers to comment on structural changes associated with FH and investigate the correlation between retinal FD and visual acuity, providing valuable insights into the relationship between retinal vascular alterations and functional vision in individuals with FH.

## 2. Materials and Methods

### 2.1. Design and Setting

This prospective study was approved by the local ethics committee of the Medical Association of Westfalen-Lippe and the University of Münster (No.: 2015-402-f-S) and adhered to the tenants of the Declaration of Helsinki. Informed consent was obtained prior to study enrollment. Patients with FH who presented at the Department of Ophthalmology, University of Muenster Medical Centre, between 1 January 2022 and 31 December 2022 as well as a healthy age- and gender-matched control group, which were retrospectively included in this study. 

### 2.2. Patient Examination

All participants of the study underwent standardized ophthalmic examination including an examination of the anterior segment, funduscopy, a refractive eye exam, and intraocular pressure measurement. Control cohort patients were required not to show any signs of ocular disease and not have a history of previous ocular surgery. Both, FH and control patients, were further required to not have history of previous retinal surgery, show any signs of media opacities that might influence retinal imaging, or suffer from diabetes mellitus. FH patients were additionally required to not have any retinal pathology other than FH, such as age-related macular degeneration (AMD). Patients were excluded from the study if their spherical equivalent was ≤−6 diopters. 

**Figure 1:**
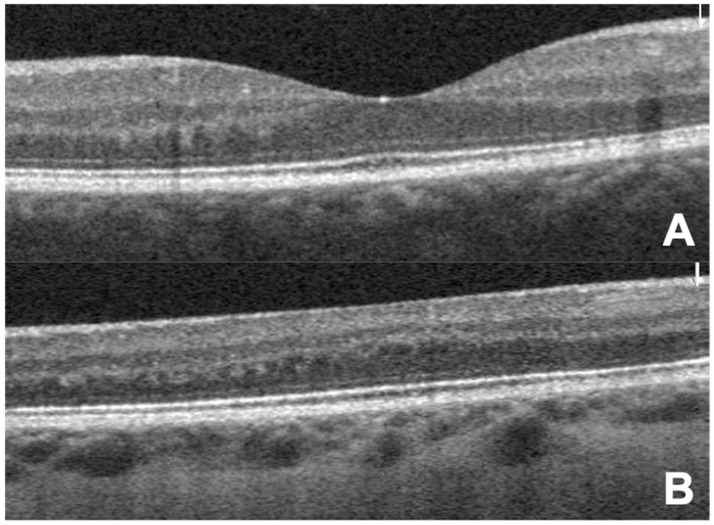
Spectral domain OCT of (**A**) a healthy retinaand (**B**) a retina with FH (Grade 4). Note the persistence of the GCL, IPL, INL and ONL in this patient.

**Figure 2:**
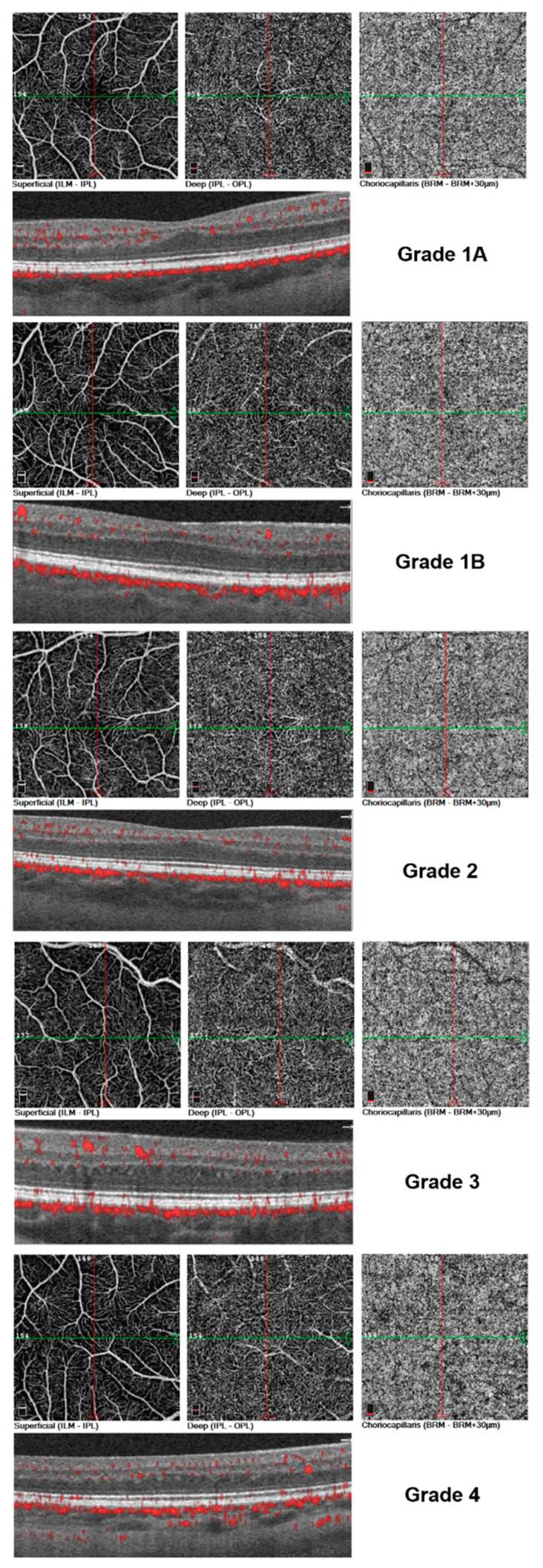
Three-by-three mm whole en face angiograms of the superficial, deep and choriocapillary plexus in eyes with different grades of FH. Upper half of each row: whole en face angiograms of different retinal layers. Bottom image of each row: corresponding structural OCT with superimposed areas of flow (in red).

The diagnosis of FH and its classification according to the Leicester Grading System for Foveal Hypoplasia [20,21] was based on the cross-sectional OCT images generated with spectral domain (SD)-OCT (Spectralis OCT; Heidelberg Inc., Heidelberg, Germany). FH patients were required to have persistence of at least two otherwise missing inner retinal layers in the fovea (ganglion cell layer (GCL), inner plexiform layer (IPL), inner nuclear layer (INL), and outer plexiform layer (ONL)) (Figure 1 and Figure 2). 

OCT-angiographic imaging was conducted directly after SD-OCT imaging under the same lighting conditions in a darkened, windowless room by a qualified examiner. Patients were required to rest for five minutes prior to OCTA imaging, in order to rule out possible effects of blood pressure and heart rate alterations [22]. Image evaluation was performed independently by two ophthalmologists experienced in OCTA imaging. Another ophthalmologist was consulted in unclear cases.

**Figure 3:**
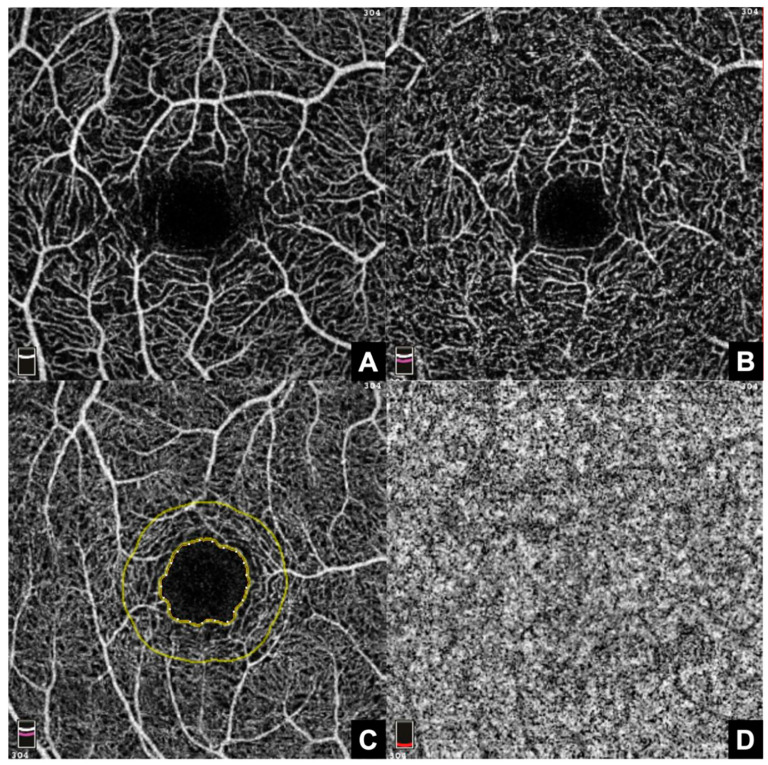
Three-by-three mm whole en face OCTA scans of a healthy retina. (**A**): Superficial capillary plexus (SCP), (**B**): deep capillary plexus (DCP), (**C**): foveal avascular zone (FAZ) outlined by the inner yellow circle, with surrounding FD-300 zone (outer yellow circle), and (**D**): choriocapillaris (CC).

OCTA imaging was conducted using the RTVue XR Avanti system (Angiovue/RTVue-XR Avanti optical coherence tomograph, Optovue Inc., Fremont, CA, USA). Eyes were imaged without topical dilatation. Angiographic imaging of the macula used 3 × 3 mm scans and generated en face representations of the inner retinal plexus, the FAZ, and choriocapillaris [Figure 3]. 

The AngioVue algorithm automatically calculated FD, which equals the ratio of bright pixels to the total number of pixels per scan and is reported as a percentage value (%), for the “whole en face” scan, representing the total FD for each retinal layer, as well as for individual sublocations within these layers. The internal segmentation algorithm distinguished between different retinal layers and placed the measurement grid over the foveal center. Identification of the foveal center in patients with FH can be challenging for imaging devices. In this trial, the OCTA device utilized an automatic tracking and follow-up system to identify the boundaries of the FAZ and track the foveal region in the eyes being imaged. However, we observed that in higher grade cases, the internal software faced difficulties accurately identifying the foveal region, leading to the misplacement of the measurement grid. To address this, we manually positioned the measurement grid above the fovea using real-time infrared images as a reference. The fovea in FH patients was then identified based on the vascular pattern seen on the angiograms, characterized by the convergence of major vessels towards the center. By following these vascular patterns, we accurately localized the foveal region in FH eyes and verified the correct localization of the measurement grid (Figure 2). If uncertainties in regard to the correct placement of the grid remained, the image was dismissed. The CC slab for each patient was semi-automatically adjusted for each scan to ensure the correct identification of Bruch’s membrane, as has been suggested previously [23].

In total, twelve parameters, each related to the macular superficial capillary plexus (SCP), the macular deep capillary plexus (DCP) and choriocapillary plexus (CC), as well as five parameters related to the FAZ were extracted and analyzed (Figure 3). The FAZ parameters included FAZ area, FAZ perimeter, and acircularity index (ACI), as well as FD-300 Area Density and Length Density, which were also calculated automatically by the internal software. The ACI represents the ratio as to which degree the morphology of the FAZ equals the symmetry of a perfect circle, with a value of 1.0 representing a perfect circle. FD-300 refers to the region in a 300 µm radius around the FAZ [24] (Figure 3).

Data on the thickness and volume of individual retinal layers were also extracted from the RTVue XR Avanti system. Total retinal thickness (RT) and total retinal volume (RV), defined as the thickness and space between the internal limiting membrane (ILM) and the retinal pigment epithelium (RPE), as well as the thickness and volume of the superficial (SP) and deep plexus (DP) were analyzed and compared between both groups. SP thickness and volume were defined as the distance and space between the nerve fiber layer (NFL) and inner plexiform layer (IPL). DP thickness and volume were defined as the distance and space between the IPL and the outer plexiform layer (OPL). We further distinguish between thickness and volume values for the entire scan (“whole”) and the central sector of the scan (“center”).

A total of 32 eyes from 16 patients diagnosed with FH, along with 32 eyes of 16 healthy, age- and gender matched individuals, underwent OCTA imaging. For each FH patient, an age- and gender-matched healthy counterpart has been recruited, ensuring that the age difference between each patient and their counterpart does not exceed 6 years. Scans displaying artifacts were excluded. To ensure imaging reliability, a quality index (QI) of ≥7 and a signal strength index (SSI) of ≥50.0 were set as thresholds for all eyes included in this study. Each macular slab was imaged at least three consecutive times using OCTA. The image with the highest QI and SSI was ultimately selected for inclusion in the subsequent statistical analysis. In cases where multiple images had the same QI and SSI, one image was randomly chosen to be drawn into the analysis. 

In all patients affected with FH, manifestation was bilateral as determined by fundoscopy and multimodal imaging including OCT and OCTA. In total six individuals with FH were excluded from the study, as OCTA imaging did not reach a quality index of ≥7 in either one of both eyes. One patient with FH fulfilled the abovementioned criteria only in one eye. Accordingly, data of the corresponding matched control patients were removed before statistical analysis was carried out. All patients ultimately included in statistical analysis had idiopathic FH without any signs for oculocutaneous albinism.

### 2.3. Statistical Analysis

Data were recorded in the spreadsheet software Microsoft Office Excel (Microsoft, Redmond, WA, USA) (2010). Data are represented as medians (interquartile range: 25% quartile; 75% quartile). 

As the normal distribution assumption could not be ensured, rank correlations were computed. However, the standard Spearman correlation coefficient does not take the clustering structure of our data into account. Hence, correspondingly adapted methods from Rosner et al. [25] with data from patients of which both eyes are available (*n* = 9) were applied. For each correlation coefficient, we also report 95% confidence intervals. No adjustments to multiple testing were made here as our analyses are of exploratory nature.

In order to account for the dependency structure, we applied corresponding rank sum test as suggested by Rosner et al. [26] and implemented by Jiang et al. [27] when comparing two groups. The matching in the recruitment process was taken into account by using a stratified version of the test and considering each patient and its matched counterpart as one stratum.

We report 95% confidence intervals that do not contain zero and *p*-values falling below 0.05 as significant findings. However, the purpose of this study is purely exploratory, and these findings should thus be treated with care or confirmed in a separate study.

Statistical analysis was performed using R version 4.2.3 [28]. The R package clusrank [27] was used to execute the rank sum tests from [26].

## 3. Results

### 3.1. Study Population Characteristics

A total of 19 eyes of 10 patients with FH as well as 19 eyes of 10 healthy, age- and gender matched controls were included in this study. Characteristics of the study population are summarized in Table 1.

Spherical equivalent (*p* = 0.47) and visual acuity (*p* = 0.70) were comparable between both groups. With the exception of one patient, both eyes of all other individuals had identical disease severity grades. In the case of the one exceptional patient, one eye had grade 1b disease and the other had grade 2 disease. QI and SSI were both noticeably greater in the control cohort than in the study group (*p* = 0.03).

### 3.2. Main Outcome: Angiographic Parameters

#### 3.2.1. Flow Density (FD)

Statistical testing revealed noticeably higher FD values in eyes with FH compared to control eyes in the foveal sectors of the SCP and DCP. The whole en face images of the SCP, DCP, and CC as well as all individual subsectors were indifferent between both cohorts (Table 2). 

#### 3.2.2. Foveal Avascular Zone (FAZ)

Evidence of noticeable differences between the groups could also be identified for the area of the FAZ, its perimeter, as well as for its symmetry, which is represented by the ACI. Characteristics of the FD-300 area were indifferent between both cohorts (Table 3). 

### 3.3. Secondary Outcome: Retinal Thickness and Volume

Total RT and RV over the entire scan area were noticeably greater in control eyes than in FH eyes. However, central RT and RV were greater in eyes with FH. Similarly, at the level of the SP, central thickness, and volume were greater in FH eyes, while at the same time the average thickness and volume of this retinal layer was reduced in comparison to control eyes. At the level of the DP, the average thickness and volume were indifferent between both cohorts, but the central sector was greater in eyes with FH than in control eyes (Table 4). 

### 3.4. Tertiary Outcome: Correlation Analysis

Correlation analysis revealed a positive correlation between VA (LogMar) and disease severity (Table 5). Note that due to the logarithmic representation of VA data in LogMar, a positive correlation between VA (LogMar) and illness severity translates into a decrease in VA with increasing disease severity. 

There was no noticeable correlation between structural FAZ parameters and disease severity (Table 5). It is important to note that due to the structure of the data and the limited sample size, correlation results should be interpreted with caution. 

## 4. Discussion

**Figure 4:**
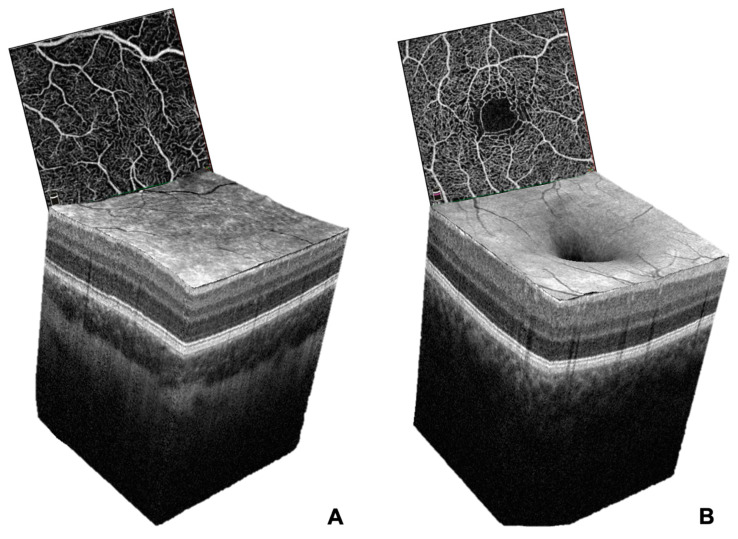
Three-dimensional reconstruction of the macular region of (**A**) an eye with FH and (**B**) a healthy retina in structural OCT imaging. The respective OCT angiograms of the SCP are displayed alongside the three-dimensional retinal models. Note that the absence of the foveal pit in the structural OCT coincides with the absence of the FAZ in the superficial whole en face OCTA image in the eye affected with FH.

OCTA has proven to be of great diagnostic value in the research of retinal pathologies [29]. To date, quantitative analysis of changes related to FH have mostly been limited to structural features, identified using histological studies or OCT [1,20,30,31]. Though the absence of a foveal pit constitutes the most striking structural feature, FH has been shown to not only be associated with structural changes, but also with vascular alterations [9,10,11,12,13,14,15,16,32] (Figure 4). 

This study extends current knowledge on retinal FD and FAZ morphology in FH and for the first time reports FD values for the CC in eyes with FH. This study is the first to report FD in all subsectors of the macular angiogram in commercially available OCTA devices and describes quantifiable differences in FAZ morphology.

FD values of the SCP, DCP, and CC were statistically indifferent between the control cohort and the FH cohort for all locations, except for the foveal sectors of the SCP and DCP. Eyes with FH had noticeably greater FD in the foveal sectors. These observations are in line with previous works [18,19]. In contrast to Chatzistergiou et al., however, whole en face FD of the DCP was not reduced in this study [18]. This difference might be attributable to the fact that Chatzistergiou et al. did not exclude patients with retinal diseases other than FH, such as AMD or diabetic retinopathy. Similar to the report of Dolz-Marco et al., the whole image FD values of the SCP and DCP did not differ noticeably in this study [14]. As Chatzistergiou et al. explain, the increase in foveal FD in both SCP and DCP can be explained by the absence of the FAZ as a result of a misdirected development of vascular tissue throughout ontogenesis [18,33].

In this study, both FAZ area and FAZ perimeter were noticeably reduced in FH eyes in comparison to healthy control eyes. Conversely, the symmetry of the FAZ was noticeably more distorted in the study group, as the ACI deviated farther from a perfect value of 1.0 in FH patients than in controls. At the same time, the density of capillaries adjacent to the FAZ (FD-300) did not differ between both groups. These angiographic observations suggest that changes in the microvasculature in FH are strictly limited to the center-most part of the fovea, involving only the inner retinal layers. 

Upon further analysis, it was revealed that the conclusion derived from the angiographic parameters could also be drawn from statistical findings of the analysis of structural thickness and volume metrics. In structural analysis, central RT and RV were increased in patients with FH in comparison to controls. The increase in thickness and volume was seen in the central sectors of the SP and DP, demonstrating that, in accordance with the changes in microvasculature, the presence of ectopic tissue in FH appears to be limited to the inner-most and central part of the fovea. Interestingly, the average total, average SP, and average DP slabs of eyes with FH were either indifferent or smaller in regard to thickness and volume in comparison to control eyes, suggesting that retinas with FH are on average thinner than normal, despite the presence of ectopic foveal layers. This observation is in line with in vivo OCT trials that have described the centripetal displacement of all retinal layers during the fetal phase to result in both cone packing and retinal thickening [9,34].

Statistical analysis revealed a negative correlation between disease severity and VA. Though we expected to see a reduced VA in the study group in comparison to the control cohort, this was ultimately not the case. As has been shown by a number of studies, VA is not necessarily decreased in FH patients [9,35,36]. Neither the presence of a foveal pit, nor of a regular FAZ appear to be the decisive features that guarantee normal visual function [9]. Contrarily, the correct anatomical formation of the outer retinal segment during the fetal period, meaning cone packing and elongation of the outer retinal layers appears to be crucial in the development of proper VA [9,20,37,38]. As described previously, the total RT of FH patients in this study was less than normal, despite indifferent thickness values in the DP and only slightly reduced thickness values in the SP, suggesting that in these patients, elongation of the outer retinal segment might not have followed a normal developmental path. As the majority of eyes (12/19) in this study had Leicester grades lower than grade 3, most eyes included did not have severe outer retinal segment changes. This might explain the similar VA between the FH cohort and the control cohort in this study.

It has previously been suggested to use OCTA as a surrogate biomarker in the grading of FH [18]. As Chatzistergiou et al. describe, they could base the diagnosis of FH on a foveal FD of >30% or an FAZ area of <0.1 mm^2^. Applying these definitions to the population in this study, we see that they quite reliably differentiate between healthy eyes and eyes with FH. In our study, the median foveal FD of the SCP was 34.39%. With the exception of only one grade 1b eye with a foveal FD of 27.28%, every other eye of the FH cohort had a FD of >30%. In contrast, the maximum foveal FD of the SCP in control eyes was 24.36%. Therefore, differentiation based on foveal SCP values appears reasonable, though in some lower grade cases, FD values alone might not be sufficient to distinguish between normal and FH. Considering the FAZ area, this parameter could also differentiate between pathological and physiological findings in this study. With the exception of one eye having an FAZ area of 0.12 mm^2^, every other eye in the FH cohort had an FAZ area of <0.90 mm^2^. 

We suggest that in addition to the parameters noted by Chatzistergiou et al., the foveal FD of the DCP, as well as the perimeter of the FAZ and the ACI could also be used to differentiate FH from healthy retinas in OCTA imaging. In this regard, ACI is of particular interest, as it is independent of the eye’s axial length [39], possibly making it more reliable than other parameters in eyes with altered retinal anatomy.

A possible correlation between OCTA parameters and the grade of FH has not been broadly covered in previous research. While there is currently a lack of information on the relationship between FD values and the grade of FH, some authors have commented on the relationship between FAZ features and disease severity. Kaidonis et al. have demonstrated progressive changes in FAZ metrics with increasing disease severity [15]. Yet, it should be noted that their case series is based on qualitative rather than quantitative changes. In contrast, we did not observe a noticeable correlation between FAZ parameters and disease severity. However, this finding should be interpreted with caution, as the small sample size of this study and the number of tied observations of disease severity limit the validity of the correlation analyses. Kaidonis et al. suggest that the observations made in OCT angiographic studies could potentially be used for the development of a grading system for FH, benefitting from the possibility to quantify changes in retinal microvascular architecture in these patients. A multi-parameter approach for diagnosing and grading FH might allow for a more detailed representation of the disease spectrum compared to previous grading systems. By incorporating quantitative data, we would anticipate an OCTA-based system to yield higher inter- and intra-observer agreement, enhancing the reliability and accuracy of FH evaluation compared to previous classification schemes. In addition, the inclusion of quantitative parameters would allow for a more objective assessment of possible changes in disease severity. This objectivity would in turn allow for the precise monitoring of disease progression over time, the tracking of future treatment responses, and the evaluation of the efficacy of potential therapies.

By providing data presented in the tables above, this study extends normative data on FD and FAZ parameters in different retinal layers and sublocations. We want to encourage other researchers to present their data in as much detail as possible in order to define diagnostic cut-off values for FH in OCTA imaging.

### 4.1. Challenges in OCTA Imaging in Eyes with FH

Of the 16 initially screened patients, 6 did not meet the inclusion criteria of this study, as the generated scans in those patients were of insufficient quality. Retinal imaging can be a challenge in patients suffering from FH or related conditions, as they might suffer from nystagmus or photophobia. We even experienced mild nystagmus to cause prominent artifacts in the final OCTA scan, making the generated image unsuitable for statistical analysis. Even if device-internal features, such as follow-up imaging or motion tracking are either activated or paused, imaging in patients with nystagmus was not feasible. Advances in internal software leading to shorter scanning times and improved motion tracking might be able to counter these current restrictions in the future.

**Figure 5:**
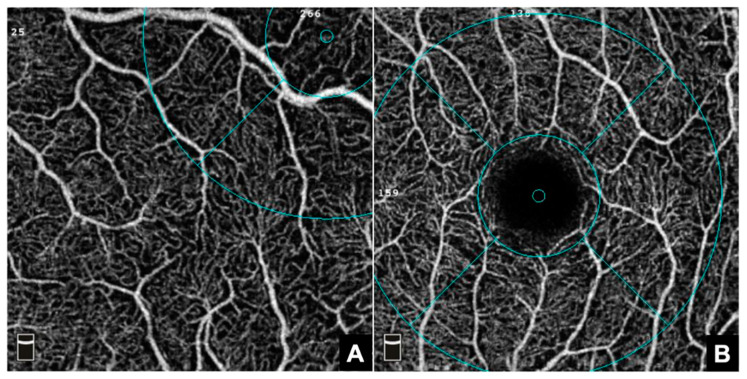
Exemplary illustration of false and correct positioning of the measuring grid (blue circle and lines) of the internal OCTA device software. (**A**): Whole en face angiogram of an eye with FH. Note that the internal software misplaced the measuring grid to a location superior-peripheral of where the FAZ would otherwise be. (**B**): Whole en face angiogram of a healthy macula with correct positioning of the internal measuring grid. Note that the foveal part of the measuring grid is correctly positioned above the FAZ.

QI and SSI were both noticeably greater in the control group than in the study group. This might as well be attributable to the more challenging image acquisition in FH patients, as the internal software of the OCTA device tracks the patient’s macula based on anatomical features. In hypoplastic foveas, the device can have reduced signal strength and overall image quality reduction, as a result of its incapability to properly reposition itself due to its incapability to correctly identify foveal structures, such as the FAZ (Figure 5).

### 4.2. Limitations

As changes in QI and SSI have been described to be associated with differences in FD values, caution is advised when comparing FD values between the two groups within this study [40,41]. However, as studies have demonstrated a positive correlation between QI/SSI and FD, it is unlikely that the difference between both groups in this regard is of significance for the outcomes presented here. This is because we have seen that foveal FD was greater in FH eyes than in controls, while featuring lesser SSI and QI values. Therefore, this indicates that the difference in absolute foveal FD between both groups might in reality be even greater than the data presented here suggest. 

The sample size of this study limits the generalizability of the results. Though this study provides a comprehensive representation of FH, as all disease grades were present among the study population, larger cohort studies are required to confirm the findings reported in this work. This heterogeneous composition of the study population at the same time limits our ability to comment on changes in angiographic, structural, and visual features in a large number of patients in a specific disease severity group. Further longitudinal cohort studies are needed here to investigate severity-specific alterations in OCTA parameters. 

## 5. Conclusions

The vascular and structural changes in FH are most pronounced in the innermost, central sector of the macula, while no noticeable differences were observed for areas further out. These changes have a negative impact on visual acuity, which worsens with increasing disease severity. Novel threshold values for FD and FAZ parameters can be used to detect and define FH in OCTA imaging. The progressive nature of alterations in the vascular architecture within the FAZ could potentially serve as a grading system for evaluating the severity of FH.

## Figures and Tables

**Table 1:** General patient characteristics. Data are presented as median (25% quartile; 75% quartile) or as absolute and relative values.

	FH	Controls
n (eyes)	19	19
n (patients)	10	10
Age (years)	21.67 (14.53; 53.16)	22.92 (19.56; 52.92)
Gender (M:F)	8:2	8:2
Study eye (R:L)	9:10	9:10
n (eyes) according to Leicester Grading System for Foveal Hypoplasia [20,21] Grade 1a Grade 1b Grade 2 Grade 3 Grade 4	2 (11%) 3 (16%) 7 (37%) 3 (16%) 4 (21%)	
Visual acuity (logMAR)	0.20 (0.15; 0.30)	0.10 (0.05; 0.20)
Spherical equivalent	0.50 (−2.44; 1.56)	−0.50 (−1.56; 1.69)
QI	7.00 (6.50; 8.00)	8.00 (7.00; 9.00)
SSI	65.70 (61.21; 70.04)	69.28 (65.55; 78.52)

FH = Foveal Hypoplasia; n = number; M = male; F = female; R = right; L = left; and logMAR = logarithm of minimum angle of resolution.

**Table 2:** Results of statistical comparison of eyes with FH to control eyes. FD values are presented as median (25% quartile; 75% quartile).

Location	Parameter	Study Group	Control Group	*p* Value
Flow Density SCP (%)	Whole en face	43.95 (40.16; 47.43)	45.87 (44.50; 48.51)	0.36
	Whole en face superior hemisphere	43.67 (39.57; 46.13)	46.07 (44.51; 47.93)	0.24
	Whole en face inferior hemisphere	44.44 (40.99; 48.51)	45.75 (44.29; 48.44)	0.52
	Fovea	34.39 (31.39; 40.47)	20.45 (16.25; 22.13)	**<0.01**
	Parafovea	45.31 (40.81; 48.73)	48.52 (47.09; 51.09)	0.29
	Parafovea superior hemisphere	45.93 (39.99; 48.47)	49.08 (46.86; 50.80)	0.21
	Parafovea inferior hemisphere	45.95 (42.34; 49.17)	48.83 (47.07; 51.25)	0.35
	Parafovea temporal	45.68 (42.42; 48.80)	47.89 (44.58; 49.86)	1.00
	Parafovea superior	45.34 (41.21; 48.65)	50.18 (48.26; 51.79)	0.07
	Parafovea nasal	44.98 (39.64; 49.06)	48.58 (45.15; 49.94)	0.49
	Parafovea inferior	45.87 (43.20; 49.86)	50.80 (47.73; 52.54)	0.24
DCP (%)	Whole en face	53.71 (49.31; 55.93)	49.45 (46.91; 54.85)	0.38
	Whole en face superior hemisphere	51.82 (48.63; 55.02)	49.91 (46.15; 54.32)	0.52
	Whole en face inferior hemisphere	54.70 (48.98; 56.25)	49.36 (47.54; 55.16)	0.36
	Fovea	53.61 (48.58; 56.52)	36.92 (31.77; 40.56)	**<0.01**
	Parafovea	53.2446.86; 55.51)	51.22 (49.08 56.12)	0.77
	Parafovea superior hemisphere	52.98 (47.33; 56.01)	51.09 (48.27; 55.83)	0.77
	Parafovea inferior hemisphere	54.24 (47.92; 55.76)	51.10 (48.81; 56.41)	0.95
	Parafovea temporal	54.35 (50.27; 56.11)	51.95 (49.49; 57.34)	0.58
	Parafovea superior	51.99 (48.81; 56.94)	51.55 (47.41; 55.30)	0.90
	Parafovea nasal	53.62 (46.52; 55.53)	50.79 (49.47; 55.62)	0.62
	Parafovea inferior	54.03 (47.82; 56.32)	50.46 (47.73; 55.52)	0.74
CC (%)	Whole en face	72.57 (68.66; 74.38)	71.74 (70.89; 72.96)	0.77
	Whole en face superior hemisphere	71.66 (68.60; 73.69)	72.17 (70.90; 72.66)	0.80
	Whole en face inferior hemisphere	71.53 (68.71; 74.99)	72.07 (70.74; 73.20)	0.88
	Fovea	70.68 (64.23; 73.71)	71.86 (67.75; 74.01)	0.31
	Parafovea	71.97(67.91; 73.70)	71.57 (70.35; 73.15)	0.79
	Parafovea superior hemisphere	71.72 (67.99; 73.38)	71.57 (70.30; 72.56)	0.54
	Parafovea inferior hemisphere	71.68 (67.83; 74.21)	72.36 (69.88; 73.56)	0.95
	Parafovea temporal	72.92 (69.81; 77.30)	74.20 (72.39; 75.55)	0.92
	Parafovea superior	71.09 (68.09; 72.94)	69.28 (67.66; 70.17)	0.09
	Parafovea nasal	71.77 (65.37; 73.56)	73.34 (71.87; 74.40)	0.28
	Parafovea inferior	72.11 (66.34; 75.48)	70.24 (67.63; 72.35)	0.43

*p* values ≤ 0.05 are highlighted in bold. SCP = superficial macular capillary plexus, DCP = deep macular capillary plexus, CC = choriocapillaris, FAZ = foveal avascular zone, mm = millimeters, ETDRS = Early Treatment Diabetic Retinopathy Study, and Parafovea = area surrounding the fovea.

**Table 3:** Results of statistical comparison of eyes with FH to control eyes. Values of FAZ parameters are presented as median (25% quartile; 75% quartile).

Location	Parameter	Study Group	Control Group	*p* Value
FAZ	FAZ area (mm^2^)	0.05 (0.03; 0.09)	0.22 (0.18; 0.27)	**0.04**
	Perimeter (mm)	0.91 (0.78; 1.24)	1.84 (1.68; 2.05)	**0.04**
	ACI	1.19 (1.14; 1.24)	1.12 (1.11; 1.15)	**0.03**
	FD-300 Area Density (%)	49.66 (44.78; 51.10)	47.96 (46.80; 49.76)	0.41
	FD-300 Area Length (%)	15.98 (13.99; 18.15)	17.30 (16.54; 18.21)	0.79

*p* values ≤ 0.05 are highlighted in bold.FAZ = foveal avascular zone, mm = millimeters, ACI = acircularity index, and FD-300 = flow density of the capillaries adjacent to the FAZ.

**Table 4:** Results of statistical comparison of retinal thickness and volume values between eyes with FH and control eyes. Data are reported for the average value over the entire scan (“whole”) and the central sector of the scan (“center”). Values are presented as median (25% quartile; 75% quartile).

Parameter	Scan Area	Location	Study Group	Control Group	*p* Value
Thickness (µm)	Whole	All (ILM-RPE)	303.20 (292.65; 313.30)	326.60 (316.90; 330.75)	**0.01**
		SP (NFL-IPL)	70.40 (67.50; 75.70)	83.00 (79.55; 87.80)	**0.01**
		DP (IPL-OPL)	66.10 (60.55; 69.40)	65.40 (63.90; 71.35)	0.92
	Center	All (ILM-RPE)	293.40 (287.15; 301.45)	265.00 (256.75; 271.10)	**0.02**
		SP (NFL-IPL)	59.30 (48.50; 61.95)	33.40 (26.00; 36.30)	**<0.01**
		DP (IPL-OPL)	56.20 (53.00; 60.45)	45.70 (44.20; 50.40)	**0.03**
Volume (mm^3^)	Whole	All (ILM-RPE)	2.73 (2.62; 2.78)	2.95 (2.87; 3.00)	**0.03**
		SP (NFL-IPL)	0.63 (0.60; 0.68)	0.75 (0.72; 0.81)	**0.03**
		DP (IPL-OPL)	0.04 (0.04; 0.05)	0.04 (0.03; 0.04)	0.45
	Center	All (ILM-RPE)	0.23 (0.22; 0.24)	0.21 (0.20; 0.21)	**0.04**
		SP (NFL-IPL)	0.04 (0.04; 0.05)	0.03 (0.02; 0.03)	**0.01**
		DP (IPL-OPL)	0.60 (0.58; 0.65)	0.60 (0.55; 0.63)	**0.03**

*p* values ≤ 0.05 are highlighted in bold. µm = micrometers; mm^3^ = cubic millimeters; SP = superficial plexus; DP = deep plexus; ILM = internal limiting membrane; RPE = retinal pigment epithelium; NFL = nerve fiber layer; IPL = inner plexiform layer; and OPL = outer plexiform layer.

**Table 5:** Estimates and confidence intervals for correlation analysis between VA (LogMar) and disease severity, as well as for correlation analysis between FAZ parameters and disease severity.

	Estimate	Lower 95% CI	Upper 95% CI
Visual acuity (LogMar)	0.78	0.11	0.97
FAZ area (mm^2^)	0.38	−0.08	0.71
Perimeter (mm)	0.45	−0.01	0.76
ACI	0.38	−0.13	0.74

CI = confidence interval, logMAR = logarithm of minimum angle of resolution, FAZ = foveal avascular zone, and ACI = acircularity index.

## Data Availability

Not applicable.
